# Changes of Coagulation and Fibrinolytic Status Detected by Thromboelastography (TEG6s^®^) in Pregnancy, Labor, Early Postpartum, Postpartum Hemorrhage and Heparin Treatment for Perinatal Venous Thrombosis

**DOI:** 10.3390/healthcare10102060

**Published:** 2022-10-17

**Authors:** Chiharu Suemitsu, Megumi Fudaba, Kohei Kitada, Yasushi Kurihara, Mie Tahara, Akihiro Hamuro, Takuya Misugi, Akemi Nakano, Masayasu Koyama, Daisuke Tachibana

**Affiliations:** 1 Department of Obstetrics and Gynecology, Graduate School of Medicine, Osaka City University, 1-4-3 Asahimachi Abeno-ku, Osaka 545-8585, Japan; 2 Department of Obstetrics and Gynecology, Graduate School of Medicine, Osaka Metropolitan University, 1-4-3 Asahimachi Abeno-ku, Osaka 545-8585, Japan; 3 Department of Obstetrics and Gynecology, Kashiwara Municipal Hospital, 1-7-9 Hozenji, Kashiwara-shi 582-0005, Japan

**Keywords:** thromboelastography, pregnancy, postpartum hemorrhage, transfusion, venous thrombosis, heparin, TEG6s^®^

## Abstract

The aims of this study are to evaluate coagulation and fibrinolytic features using TEG6s^®^ in normal pregnant courses, in the early postpartum period and in cases with postpartum hemorrhage (PPH) caused by uterine atony. We also analyze cases with deep venous thrombosis (DVT) and/or pulmonary embolism (PE) under treatment with unfractionated heparin. The non-pregnant women (*n* = 13) and healthy pregnant women (at 9–13 weeks of gestation (*n* = 13), at 27–30 weeks of gestation (*n* = 14), at 35–38 weeks of gestation (*n* = 14)) were cross-sectionally studied, while the normal pregnant women at delivery (*n* = 14) were sequentially investigated. Blood samples from those patients with PPH (*n* = 15) and DVT and/or PE (*n* = 11) were also obtained and compared with those of normal women. Significant changes of clot formation parameters were observed in all parameters and, interestingly, fibrinolytic parameter (LY30) was maintained at a low value even within 120 min after placental delivery (median of LY30; 0) and also in cases with uterine atony (median of LY30; 0.1). The parameter that indicates the effectiveness of heparin showed strong correlation (R = 0.788) with activated partial thromboplastin time. Thromboelastography may be less sensitive to fibrinolysis in the conditions of uterine atonic bleeding.

## 1. Introduction

Thromboelastography is a simple, rapid and accurate system for analyzing coagulopathy using whole blood at the bedside, and it has been widely used in the critical care of emergent situations all over the world [1,2,3]. This technique has recently been revisited due to its ability to provide information on multiple stages of hemostasis (clot formation, stabilization, and lysis), from routine coagulation tests to a variety of other, more sophisticated analyses [4,5]. The advantages of thromboelastography are preferred to be applied to the fields of liver transplantation, cardiac surgery, and trauma and to serve as guide for transfusion protocols [6,7,8,9].

Pregnancy, however, is an ambivalent condition where women are occasionally faced with postpartum hemorrhage (PPH), which occurs around the time of placental removal, and an increased risk of deep venous thrombosis (DVT) and/or pulmonary embolism (PE) caused by enhanced production of coagulation factors, vascular dilatation, and restricted venous return by the gravid uterus as a result of maternal physiological changes. [5,10]. In the obstetrical practice, the incidence of PPH tends to increase in many developed countries [11,12]. DVT and PE are raised as leading causes of pregnancy-related deaths, and in cases where unstable and/or newly developed DVT and/or PE are recognized, immediate anticoagulant therapy should be started and continued just before the initiation of delivery [13,14]. Furthermore, amniotic fluid embolism, which is relatively rare but characterized by excessive fibrinolysis and resulting catastrophic coagulopathy, with a possible anaphylactoid reaction, may occur in uncomplicated pregnant women during or immediately after delivery [15,16,17].

Some studies have reported on the usefulness of thromboelastography as a tool in which to evaluate coagulative conditions and to guide obstetricians toward prompt treatment. The TEG6s^®^ system (Hemostasis System, Boston, MA, USA), which is newly developed, enables four different assays simultaneously from a single blood sample: CK (citrated kaolin), CRT (citrated rapidTEG), CKH (citrated kaolin with heparinase) and CFF (citrated functional fibrinogen). Among them, CKH contains kaolin and heparinase to neutralize the effects of heparin in the blood. The time to initial clot formation, or R time, of CK will be longer than the R time of CKH in a blood sample with heparin [18]. The TEG6s^®^ device has been found to have excellent repeatability within device (CK-R: 0.9, CFF-MA: 0.99, CRT-MA; 0.95, CRT-MA; 0.99 and CK-LY30; 0.88) [3]. The TEG6s^®^ system will help clinicians to manage PPH patients and to monitor anti-coagulant therapy during pregnancy and postpartum; however, only limited papers on the subject have been published so far [5,10]. 

The aims of this study are to evaluate coagulation and fibrinolytic features using TEG6s^®^ in normal pregnant courses, in the early postpartum period and in cases with PPH. We also analyze cases with DVT and/or PE under treatment with heparin.

## 2. Materials and Methods

### 2.1. Collection of Patients

Uncomplicated women with a singleton pregnancy who were managed and delivered at Osaka Metropolitan University Hospital were included in this study from October 2020 to February 2022. The study was approved by the institutional review board (Approved Number: 2020-244), and all patients gave their informed written consent before inclusion. 

**Figure 1:**
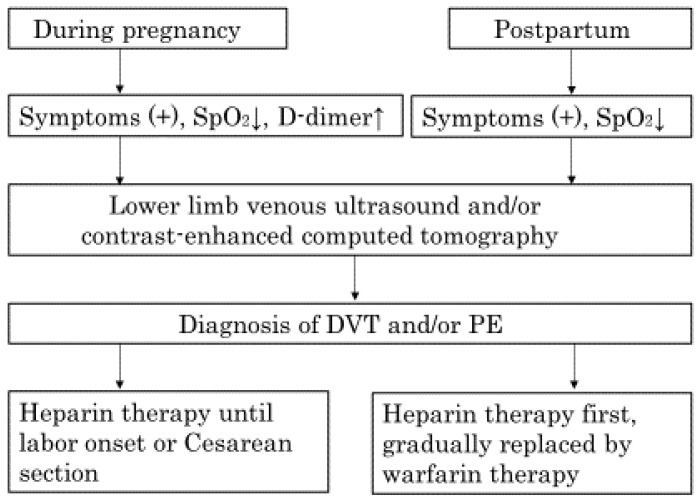
A flow diagram showing the process of diagnosis and treatment of patients with DVT and/or PE. Symptoms include dyspnea, chest pain and leg pain etc. SpO_2_ = peripheral oxygen saturation, DVT = deep venous thrombosis, PE = pulmonary embolism.

Additional venous blood draws were performed at the timing of a routine schedule: 1st trimester; 9–13 weeks of gestation, early 3rd trimester; 27–30 weeks of gestation, and mid-3rd trimester; 35–38 weeks of gestation. Sequential venous blood samples were also obtained at the timing of delivery from patients without PPH (estimated blood loss (EBL) <500 mL; during 1st stage of labor and 15, 30, 60, and 120 min after placental delivery). The blood samples of PPH patients due to uterine atony (EBL of ≥2000 mL) were obtained within 2 h after placental delivery and before the start of any interventions such as transfusion and/or uterine artery embolization. The blood loss was estimated with the weight of the postpartum-pad and gauze. Control samples were also taken from non-pregnant healthy women. All data of the non-pregnant women and pregnant women were collected cross-sectional, while the data of the women at delivery were collected sequential. We also obtained blood samples from those patients with DVT and/or PE and who were treated with unfractionated heparin before and/or after delivery. Once patients were diagnosed with DVT and/or PE, treatment with continuous heparin infusion was initiated with monitoring to ensure that activated partial thromboplastin time (APTT) values were 1.5 to 2.5 times with control values. When APTT values reached targeted values, heparin dosing was determined and then patients were switched to subcutaneous heparin injection at the same dose. Figure 1 shows a flow diagram for the diagnosis and treatment of the patients with DVT and/or PE. We excluded patients who developed hypertensive disorders in pregnancy or gestational diabetes mellitus during pregnancy. 

### 2.2. Thromboelastography Assay

**Figure 2:**
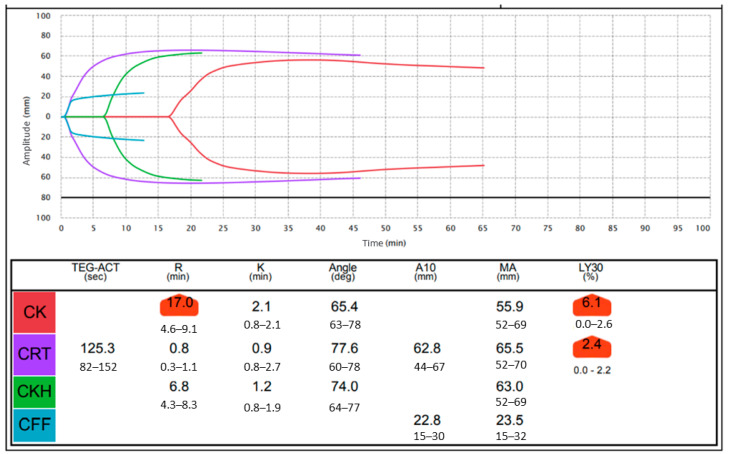
A typical trace of TEG6s^®^ parameters in a patient who developed deep venous thrombosis at 12 weeks of gestation and started heparin therapy; the red line is citrated kaolin (CK), the purple line is citrated rapidTEG (CRT), the green line is citrated kaolin with heparinase (CKH), and the blue line is citrated functional fibrinogen (CFF). The difference between CK and CKH clearly shows the effect of heparin administration. R = reaction time, K = clot formation time, Angle = alpha angle, MA = maximum amplitude, LY30 = lysis at 30 min.

Blood samples were collected by peripheral venipuncture of an upper limb and analyzed by TEG6s^®^. Briefly, whole blood (2 mL) was collected in anticoagulation tubes containing 3.2% sodium citrate, and the sodium citrate was mixed thoroughly with the sample before testing. The test cartridges were kept refrigerated and the samples were kept at room temperature until inspection. As a rule, testing was performed within 2 h of blood collection, but in most samples were tested within a few minutes because there were two TEG6s^®^ machines in our delivery rooms. In this case, 300 µg of the collected blood was injected into the test cartridge using the pipette provided. Among the thromboelastographic parameters, reaction time (R) is the time from the start of analysis until the thrombus amplitude reaches 2 mm and reflects the adequacy of coagulation factors. Clot formation time (K) is the time from the thrombus amplitude reaching 2 mm until it reaches 20 mm, and this reflects clot kinetics. The alpha angle (Angle) is the slope of a tangent line from the tracing at the midpoint between R and K and reflects the fibrinogen level. Maximum amplitude (MA) is the absolute thrombus strength and reflects platelet function, and clot lysis at 30 min (LY30) reflects clot lysis [5]. The CK (citrated kaolin) assay is a kaolin activated test method which is used to reduce the variability and running time of a native whole blood sample. The CRT (citrated rapidTEG) assay contains tissue factor and kaolin to be reached more quickly. CKH (citrated kaolin with heparinase) contains kaolin and heparinase to neutralize the effects of heparin in the blood. The R time of CK will be longer than the R time of CKH in a blood sample with heparin. The CFF (citrated functional fibrinogen) assay inhibits platelet aggregation by excluding the contribution to clot strength and measures the contribution of fibrinogen to clot strength [18]. Figure 2 shows a sample of a TEG6s^®^ thromboelastographic parameter trace of a patient who developed DVT at 12 weeks of gestation and started heparin therapy; the red line is CK, the purple line is CRT, the green line is CKH, and the blue line is CFF. The difference between CK and CKH clearly shows the effect of the heparin administration. We defined the difference between R of CK and R of CKH as delta R (delta R = R of CK—R of CKH). For the measurement of APTT, Levohem APTT-SLA (Sysmex Corporation, Kobe, Japan) was used as the measuring reagent and Sysmex CN-6000 (Sysmex Corporation, Kobe, Japan) was used as the coagulation meter.

### 2.3. Statistical Analysis

Statistical data were processed and analyzed using the software SPSS (version 24.0). All data were expressed as median (range) and *n* (%) and tested using the Kruskal-Wallis test. Statistical comparisons between the two groups were tested with the Mann-Whitney test. Differences in results were considered statistically significant when *p* < 0.05. Correlations with R values above 0.7 were considered as a strong correlation.

## 3. Results

### 3.1. Patient Enrollment

We recruited cross-sectional, 13 healthy, non-pregnant women (NP group) and 47 uncomplicated pregnant women, 15 patients with PPH (PPH group), and 11 patients with DVT and/or PE (DVT/PE group). Six uncomplicated pregnant women were excluded because of subsequential revealed hypertensive disorder in pregnancy (*n* = 4) and gestational diabetes mellitus (*n* = 2). Finally, blood samples were collected from 13 cases at 9–13 weeks of gestation (9–13 GW group), 14 cases at 27–30 weeks of gestation (27–30 GW group), and 14 cases at 35–38 weeks of gestation (35–38 GW group).

On the other hand, 14 women agreed with sequential blood collection during and after delivery. Some tests could not be performed in the delivery group because thromboelastography testing was not performed within 2 h after blood collection or because blood samples were not obtained. Finally, 8 women were included in the 1st stage of labor group (Labor group), 14 women in the 15 min after placental delivery group (15 min group), 11 women in the 30 min after placental delivery group (30 min group), 14 women in the 60 min after placental delivery group (60 min group), and 14 women in the 120 min after placental delivery group (120 min group).

Table 1 shows the characteristics of the study population. The median number of weeks of delivery was 39 weeks in the delivery group and 38 weeks in the PPH group. All postpartum cases were vaginal deliveries. There were no significant differences in age, pre-pregnancy body mass index, or nulliparous rate between the non-pregnant, pregnant, delivery, PPH and DVT/PE groups.

### 3.2. Comparison of Thromboelastographic Parameters in Different Groups

Table 2 shows the results of thromboelastographic parameters in CK assay obtained with the TEG6s^®^ system. Significant changes were observed in all parameters and, interestingly, LY30 was maintained at a low value even within 120 min after placental delivery and also in cases with PPH. In addition, thromboelastographic parameters in CRT, CKH and CFF assay are shown in Appendix A (Table A1).

**Figure 3:**
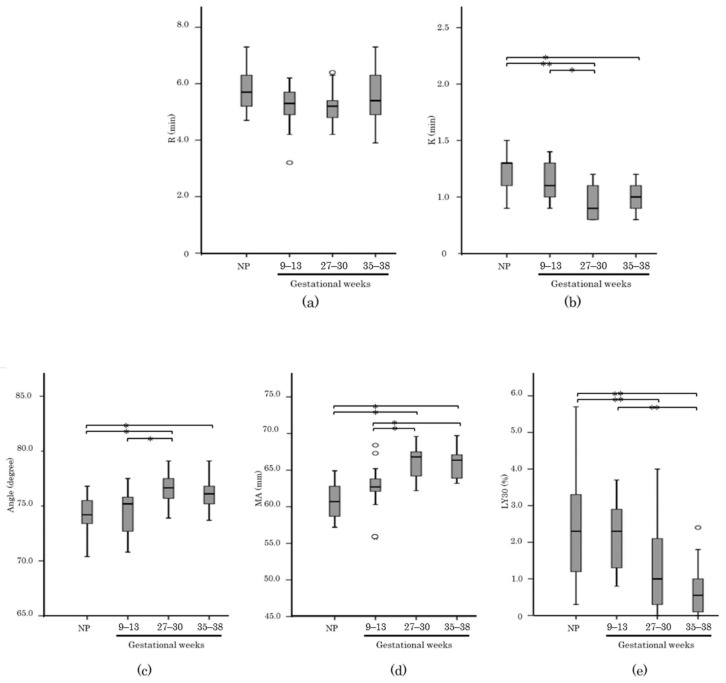
Box-and-whiskers plots showing the results of thromboelastographic parameters; R (**a**), K (**b**), Angle (**c**), MA (**d**), LY30 (**e**) in CK assays between pregnancy and non-pregnant woman (NP). The band inside the box shows the median. The Mann-Whitney test was performed to calculate *p* values (*: *p* < 0.05, **: *p* < 0.001).

**Figure 4:**
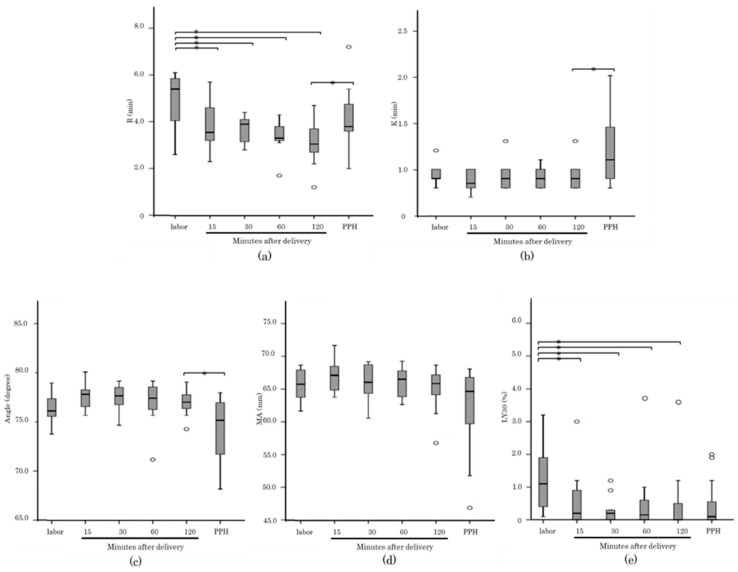
Box-and-whiskers plots showing the results of thromboelastographic parameters; R (**a**), K (**b**), Angle (**c**), MA (**d**), LY30 (**e**) in CK assays between 1st stage of labor, postpartum and postpartum hemorrhage (PPH). The band inside the box shows the median. The Mann-Whitney test was performed to calculate the *p* values (*: *p* < 0.05).

Figure 3 and Figure 4 shows a comparison of the thromboelastographic parameters of the CK assay in each group. There were significant changes in K, Angle, MA and LY30 between the NP group and pregnant women at 27–30 GW and 35–38 GW. Furthermore, R and LY30 of postpartum periods showed a significant shortening compared with those in the 1st stage of labor group. The PPH cases showed a significant increase in R and K and a decrease in Angle. However, there was no significant difference in MA and LY30 compared to the data of the normal delivery group (120 min after placental delivery). Fibrinogen level measured by the Claus method showed moderate correlation with MA (R = 0.697).

### 3.3. Correlation between Thromboelastographic Parameters and APTT Ratio in the Patients with Heparin Therapy

**Figure 5:**
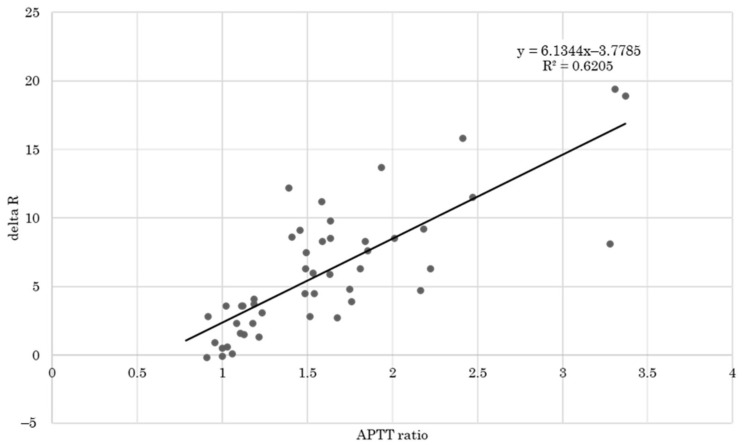
Correlation analysis between delta R (difference between R of CK and R of CKH) and the activated partial thromboplastin time (APTT) ratio. CK = citrated kaolin, CKH = citrated kaolin with heparinase.

As shown in Figure 5, linear regression indicates a strong correlation (R = 0.788) between the delta R and the APTT in pregnant patients with DVT and/or PE. 

## 4. Discussion

### 4.1. Main Findings and Importance

We observed the decrease of K, and LY30 and the increased Angle and MA throughout pregnancy before labor onset. In addition, compared with data during pregnancy, clot formation was further strengthened, and clot lysis stayed at low activity during labor and in the early postpartum period. In the PPH group, clot formation was shown to be delayed by the increase of R and K, and by the decrease of Angle, although the strength of the clot was maintained, as shown by unchanged MA and LY30. This observation suggests that the coagulopathy in this setting with atonic bleeding may appear in delayed R and K and decreased Angle caused by shortening of coagulation factors except fibrinogen and/or platelet counts. In the DVT/PE group, delta R showed a significantly strong correlation with the APTT ratio, thus suggesting that delta R well reflects the effectiveness of heparin therapy. As such, we firstly demonstrated coagulation and fibrinolysis specificity using TEG6s^®^ in normal pregnant women, early postpartum, and PPH cases in Japanese population. 

### 4.2. Comparison of TEG6s^®^ and TEG5000^®^

Using resonance-frequency elasticity measurements and a disposable multichannel microfluidic cartridge, TEG6s^®^ was designed to reduce the technical requirements of pipetting the blood sample, and vulnerability to vibration of TEG5000^®^, and to offer more reliable data for the healthcare providers at emergent critical care [3]. Before the advent of TEG6s^®^, some researchers investigated the hemostatic parameters using TEG5000^®^ (introduced in clinical practice in 1976) in the 3rd trimester of normal pregnancy, and the enhancement of clot formation was observed in all reports [10,19,20]. There is only one study on changes in clot formation during pregnancy, and that paper reported that clot formation became stronger as the gestational week progressed [19]. These results are consistent with ours, which were obtained with the TEG6s^®^ system. As to the fibrinolytic parameter, however, there remains some room for verification. Since fibrinolytic steps are expected to change with time after placental delivery and seem to greatly affect the results, the timing of blood sampling should be carefully determined [21,22]. According to previous papers, in fact, postpartum blood collections are drawn soon after cesarean delivery, 6 h after vaginal delivery, and between 12 to 24 h after cesarean delivery, and these might be somehow indicated by the incompatible results between the studies [10,19,20]. In this sense, our data on sequential monitoring of fibrinolysis in the immediate postpartum period provide essential information in that they revealed fibrinolytic changes and trends within the first 2 h postpartum, when PPH occurs most frequently.

### 4.3. Usefulness of TEG6s^®^ for PPH 

Based upon the data at the level of bleeding over 2000 mL, Karlsson et al. examined TEG5000^®^ parameters by blood loss volume in PPH cases. Based upon their observation that Angle and MA significantly decreased, they reported that impaired hemostasis was observed in cases with a blood loss of more than 2000 mL [23]. More recently, McNamara et al. reported that the data of PPH women, using the data obtained with ROTEM^®^ Delta (Werfen, Barcelona, Spain). They demonstrated that women with placental abruption with severe coagulopathy required higher doses of fibrinogen concentrate than women with other causes [24]. Furthermore, Bell et al. reported that in cases with placental abruption, there was no difference in the rate of hypofibrinogenemia between cases with blood loss of 1500–2500 mL and those with blood loss of more than 2500 mL [25]. Their observation suggested that the amount of blood loss did not predict the requirement for fibrinogen replacement in the settings of placental abruption, and that thromboelastometry might be useful as an indicator of fibrinogen replacement therapy. In a meticulously investigated study, ROTEM^®^ Sigma (Werfen, Barcelona, Spain), a fully automated successor, has also been introduced and reported to correlate with Claus fibrinogen [26]. As for TEG5000^®^ guided resuscitation, Hurwich et al. reported a case in which the management protocol of massive hemorrhage in non-obstetrical fields, such as cardiac surgery and trauma, was applied and that the patient was successfully revived after cardiac arrest [27,28,29]. Although data for PPH assessed with thromboelastography have been gradually accumulating, there are still few reports following a thromboelastography-guided transfusion protocol [5,23,24,25,26,27,28,29]. Furthermore, many cases appear to have been treated empirically, probably because normal values for the early postpartum period have not yet been established and because there is no transfusion protocol specialized for PPH. In terms of precise assessment and prompt differential diagnosis, our meticulous results, including the fibrinolytic assessment throughout pregnancy and during the early postpartum period, might provide helpful information for the treatment of PPH.

### 4.4. Usefulness of TEG6s^®^ for Monitoring the Effect of Heparin

Owing to maternal physiological changes, such as the enhanced production of coagulation factors, vascular dilatation, and restricted venous return by the gravid uterus, venous thromboembolism is a major cause of maternal morbidity and mortality [13,14]. If a thrombus is detected and the patient is considered at a high risk for pulmonary embolism, anticoagulation therapy may be initiated and continued until just before the start of labor. APTT is widely used for the evaluation of the heparin effect, although caution is required because monitoring by APTT alone may promote bleeding tendency due to excessive heparin administration [30,31]. Previous studies have suggested that the heparin dosage might be reduced by monitoring with thromboelastography during extracorporeal membrane oxygenation (ECMO) and dialysis, based on the observations that the R of thromboelastography reflects the efficacy of heparin in ECMO, whereas APTT mainly reflects heparin concentration of the blood samples [32,33]. We firstly revealed that delta R has a strong correlation with the APTT ratio in pregnant and early postpartum cases with DVT and/or PE. Further studies are needed to elucidate whether thromboelastography is more useful than APTT for monitoring heparin administration in pregnancy, where coagulation factors show dynamic enhancement. Our data suggested that delta R could replace APTT and/or anti-Xa assay, however further study should be performed to prove our hypothesis in pregnant women.

### 4.5. Study Limitations and Strength

The limitations of the present study are that the small number of the patients for each group and the data of the pregnant women were collected cross-sectional, and that we did not compare TEG6s^®^ to other devices (TEG5000^®^ and ROTEM^®^ Sigma, etc.), and that we did not assess thromboelastographic parameters in cases with placental abruption or amniotic fluid embolism, where life-threatening coagulopathy rapidly occurs. Furthermore, thromboelastography may be less sensitive to fibrinolysis, except in conditions of excessive hyperfibrinolysis [17,34,35]. We also did not show the superiority of thromboelastography to APTT for monitoring the effect of heparin in cases with thrombus. The genetic background for DVT has been reported to vary from ethnicity. Protein S, protein C and antithrombin deficiency are the major DVT risk factors in Japanese [36]. On the other hand, the factor V Leiden mutation and the prothrombin G20210A mutation are widely distributed among Caucasians, with 30–60% of pregnant women with thrombosis having these mutations [37,38]. Thus, there remains the possibility that our results should be interpreted in light of ethnicity and region. However, the strengths of our study are that the data were prospectively obtained and firstly revealed the sequential changes of hemostasis and fibrinolysis during pregnancy and in the early postpartum period when PPH occurs most frequently.

## 5. Conclusions

This is the first study to evaluate the coagulation and fibrinolytic feature using TEG6s^®^ in normal pregnant courses, in the early postpartum period and in cases with PPH. Our data of thromboelastographic parameters system will allow us to improve comprehension coagulation and fibrinolytic statuses. We believe that the present study helps to explore the pathology of PPH, and our newly demonstrated data will encourage us to initiate prompt and individualized treatment for coagulopathy. 

## Figures and Tables

**Table 1:** Characteristics of the study population.

	NP *n* = 13	9–13 GW *n* = 13	27–30 GW *n* = 14	35–38 GW *n* = 14	Delivery *n* = 14	PPH *n* = 15	DVT/PE *n* = 11	*p*
Age (years)	30.0 (27–39)	33.0 (29–42)	31.0 (25–42)	33.0 (28–39)	32.0 (26–41)	36.0 (26–44)	30.0 (27–42)	0.382
Pre-pregnancy BMI (kg/m^2^)	20.0 (18.5–22.0)	20.1 (15.9–32.9)	21.4 (15.8–33.6)	21.1 (17.1–32.5)	20.9 (16.7–26.0)	22.2 (19.2–26.2)	22.0 (18.0–31.6)	0.689
Nulliparity, *n* (%)	54	54	50	21	64	40	36	0.213
EBL at TEG sampling (mL)					295 (75–478)	2520 (2000–3885)		
EBL total (mL)					295 (75–478)	2985 (2000–6000)		

Data are median (range) or number. NP = non-pregnant woman, GW = weeks of gestation, PPH = postpartum hemorrhage, DVT/PE = deep venous thrombosis/pulmonary embolism, BMI = Body Mass Index, EBL = estimated blood loss.

**Table 2:** The results of thromboelastographic parameters in CK assay obtained with TEG6s^®^.

	NP *n* = 13	9–13 GW *n* = 14	27–30 GW *n* = 14	35–38 GW *n* = 14	Labor *n* = 8	15 min *n* = 14	30 min *n* = 11	60 min *n* = 14	120 min *n* = 14	PPH *n* = 15	*p*
R (min)	5.7 (4.7–6.7)	5.3 (4.2–6.2)	5.2 (4.2–6.4)	5.4 (4.1–7.3)	5.6 (2.6–6.8)	3.55 (2.3–5.7)	3.9 (2.8–4.4)	3.3 (1.7–4.3)	3.05 (1.2–4.7)	3.8 (2.0–7.2)	<0.001
K (min)	1.3 (0.9–1.5)	1.1 (0.9–1.4)	0.9 (0.8–1.2)	1.0 (0.8–1.2)	0.9 (0.8–1.2)	0.85 (0.7–1)	0.9 (0.8–1.3)	0.9 (0.8–3.4)	0.9 (0.8–1.3)	1.1 (0.9–2.8)	<0.001
Angle (degree)	74.2 (70.4–76.8)	75.2 (70.8–77.5)	76.65 (73.9–79.1)	76.1 (50.6–79.1)	76.0 (73.7–79)	77.85 (75.7–80.1)	77.7 (74.7–79.2)	77.45 (71.2–79.2)	77.05 (74.3–79.1)	75.2 (63.7–78.0)	<0.001
MA (mm)	60.7 (57.2–64.9)	62.7 (55.7–68.4)	66.8 (62.2–69.6)	66.35 (63.2–69.7)	66.1 (61.4–68.7)	67.15 (63.8–71.7)	66.1 (60.6–69.2)	66.55 (42.6–69.3)	65.9 (56.8–68.7)	64.7 (46.9–68.1)	<0.001
LY30 (%)	2.3 (0.3–5.7)	2.3 (0.8–3.7)	1.0 (0–3.1)	0.55 (0–2.4)	1.0 (0–3.2)	0.2 (0–1.2)	0.2 (0–1.2)	0.15 (0–3.7)	0 (0–3.6)	0.1 (0–1.9)	<0.001

Data are median (range). Since CK assay is the standard method in thromboelastograpy, the results in CK assay are shown in this table and those in the other assays are shown in the Appendix A (Table A1). CK = citrated kaolin, R = reaction time, K = kinetic time, Angle = alpha angle, MA = maximum amplitude, LY30 = percent lysis at 30 min, NP = non-pregnant woman, GW = weeks of gestation, labor = 1st stage of labor, PPH = postpartum hemorrhage.

## Data Availability

All data related to this study are contained within the manuscript.

## Appendix A

**Table A1:** The results of thromboelastographic parameters in CRT, CKH and CFF assay obtained with TEG6s^®^.

	NP *n* = 13	9–13 GW *n* = 14	27–30 GW *n* = 14	35–38 GW *n* = 14	Labor *n* = 8	15 min *n* = 14	30 min *n* = 11	60 min *n* = 14	120 min *n* = 14	PPH *n* = 15	*p*
**CRT**											
TEG-ACT	97.3 (87.9–116)	87.9 (69.2–125.3)	78.5 (69.2–116)	78.5 (49.2–87.9)	83.2 (69.2–97.3)	69.2 (69.2–97.3)	78.5 (69.2–97.3)	73.9 (69.2–97.3)	73.9 (69.2–97.3)	87.9 (69.2–209.5)	<0.001
R (min)	0.5 (0.4–0.7)	0.4 (0.2–0.8)	0.3 (0.2–0.7)	0.3 (0.2–4.7)	0.4 (0.2–0.5)	0.2 (0.2–0.5)	0.3 (0.2–0.5)	0.3 (0.2–0.5)	0.3 (0.2–0.5)	0.4 (0.2–1.7)	<0.001
K (min)	1.5 (1.0–1.9)	1.3 (0.8–1.6)	0.9 (0.8–1.3)	0.9 (0.8–6.4)	1.0 (0.8–1.3)	0.9 (0.8–1.3)	0.9 (0.8–2.3)	1.0 (0.8–2.6)	1.0 (0.8–1.3)	1.4 (1.0–3.6)	<0.001
Angle (degree)	71.7 (69.3–76.9)	74.7 (73.0–80.6)	77.6 (75.4–80.0)	77.8 (45.8–79.6)	77.6 (75.3–80.0)	77.7 (75.5–79.6)	77.8 (76.2–79.2)	77.4 (72.9–79.6)	77.5 (73.8–78.5)	74.1 (56.1–78.1)	<0.001
A10	55.5 (50.7–64.5)	60.2 (47.2–69.3)	61.9 (58.4–67.9)	63.1 (23.4–68.9)	63.8 (57.7–66.9)	64.0 (59.3–68.9)	64.4 (44.0–67.6)	60.9 (36.3–67.2)	60.7 (43.3–65.6)	57.4 (32.9–65.1)	<0.001
MA (mm)	61.1 (57.5–67.5)	64.1 (54.3–70.2)	65.9 (63.6–68.4)	66.3 (30.6–70.3)	67.2 (63.0–68.9)	67.5 (64.3–70.4)	67.0 (55.9–69.3)	66.3 (48.8–69.3)	65.7 (53.4–68.3)	63.1 (44.2–67.3)	<0.001
LY30 (%)	1.8 (0.3–6.3)	1.7 (0.1–3.5)	0.4 (0–2.2)	0 (0–0.9)	0.2 (0–3.2)	0.05 (0–3.2)	0 (0–0.7)	0 (0–2.7)	0 (0–2.9)	0 (0–1.9)	<0.001
**CKH**											
R (min)	5.4 (4.7–6.8)	5.6 (3.4–6.5)	5.2 (4.3–7.0)	5.2 (3.8–7.4)	4.7 (2.5–6.2)	3.6 (2.2–5.8)	3.7 (2.7–4.2)	3.3 (1.5–4.5)	3.2 (1.2–3.7)	4.2 (2.0–5.2)	<0.001
K (min)	1.1 (0.9–1.6)	1.1 (0.9–1.5)	0.9 (0.8–1.3)	1.1 (0.9–6.0)	1.0 (0.8–1.3)	0.9 (0.8–1.0)	0.9 (0.8–1.5)	0.9 (0.8–2.7)	0.9 (0.8–1.4)	1.1 (0.8–2.8)	<0.001
Angle (degree)	74.8 (69.8–77.0)	75.2 (72.9–77.6)	76.3 (72.4–78.4)	75.9 (49.9–78.0)	77.2 (72.8–79.5)	77.5 (75.3–79.8)	77.4 (72.9–79.0)	77.1 (72.2–80.1)	77.1 (74.8–77.9)	75.2 (62.3–78.4)	<0.001
MA (mm)	61.9 (56.7–65.8)	62.7 (55.0–68.9)	66.4 (61.8–67.9)	66.4 (29.4–70.1)	65.9 (62.3–68.5)	66.5 (62.4–69.7)	66.4 (58.2–69.1)	66.2 (45.9–69.6)	65.8 (58.5–69.0)	64.1 (46.9–67.9)	<0.001
**CFF**											
A10	18.6 (16.7–25.3)	20.3 (14.4–37.4)	25.3 (20.1–34.4)	26.4 (22.0–32.9)	23.6 (20.3–28.9)	24.6 (20.4–30.3)	25.1 (20.1–29.1)	24.7 (16.4–30.3)	25.4 (19.1–29.3)	19.3 (2.9–23.9)	<0.001
MA (mm)	19.2 (17.4–27.7)	20.2 (15.6–39.5)	25.7 (20.4–37.1)	27.3 (21.1–35.2)	24.6 (20.3–31.1)	26.4 (20.5–34.5)	26.2 (20.2–32.1)	25.6 (16.4–33.4)	26.6 (19.1–32.4)	19.5 (2.9–24.6)	<0.001

Data are median (range). CRT = citrated rapidTEG, CKH = citrated kaolin with heparinase, CFF = citrated functional fibrinogen, R = reaction time, K = kinetic time, Angle = alpha angle, MA = maximum amplitude, LY30 = percent lysis at 30 min, NP = non-pregnant woman, GW = weeks of gestation, labor = 1st stage of labor, PPH = postpartum hemorrhage.
